# A Two-Stream Method for Human Action Recognition Using Facial Action Cues

**DOI:** 10.3390/s24216817

**Published:** 2024-10-23

**Authors:** Zhimao Lai, Yan Zhang, Xiubo Liang

**Affiliations:** 1 School of Immigration Administration (Guangzhou), China People’s Police University, Guangzhou 510663, China; laizhimao@cppu.edu.cn; 2 School of Immigration Administration, China People’s Police University, Langfang 065000, China; zhangyan@cppu.edu.cn

**Keywords:** human action recognition, deep learning, facial action, fine-spatio-multitemporal, normalized temporal attention

## Abstract

Human action recognition (HAR) is a critical area in computer vision with wide-ranging applications, including video surveillance, healthcare monitoring, and abnormal behavior detection. Current HAR methods predominantly rely on full-body data, which can limit their effectiveness in real-world scenarios where occlusion is common. In such situations, the face often remains visible, providing valuable cues for action recognition. This paper introduces Face in Action (FIA), a novel two-stream method that leverages facial action cues for robust action recognition under conditions of significant occlusion. FIA consists of an RGB stream and a landmark stream. The RGB stream processes facial image sequences using a fine-spatio-multitemporal (FSM) 3D convolution module, which employs smaller spatial receptive fields to capture detailed local facial movements and larger temporal receptive fields to model broader temporal dynamics. The landmark stream processes facial landmark sequences using a normalized temporal attention (NTA) module within an NTA-GCN block, enhancing the detection of key facial frames and improving overall recognition accuracy. We validate the effectiveness of FIA using the NTU RGB+D and NTU RGB+D 120 datasets, focusing on action categories related to medical conditions. Our experiments demonstrate that FIA significantly outperforms existing methods in scenarios with extensive occlusion, highlighting its potential for practical applications in surveillance and healthcare settings.

## 1. Introduction

**Figure 1:**
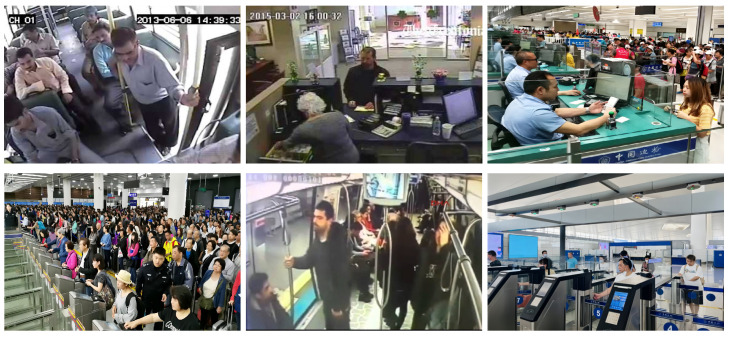
Surveillance cameras are installed indoors and in vehicles to capture clear images of people’s faces. In these public settings, body occlusion occurs frequently.

**Figure 2:**
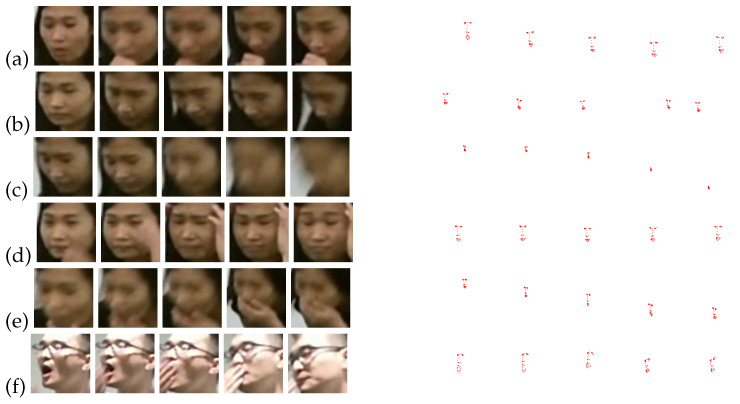
Samples of facial image sequences and landmark sequences in NTU RGB+D 120. (**a**) sneeze/cough, (**b**) staggering, (**c**) falling down, (**d**) headache, (**e**) nausea/vomiting, and (**f**) yawn.

As one of the hotspots in computer vision studies, human action recognition is leveraged in a variety of application scenarios, including video surveillance, healthcare monitoring, and abnormal behavior recognition. Current approaches predominantly utilize full-body data for action recognition, often overlooking the issue of significant occlusion [1]. We argue that these methods may lack robustness in real-world surveillance scenarios where occlusion is prevalent. For instance, as depicted in Figure 1, while the human body may be partially obscured, the face typically remains visible due to the positioning of surveillance cameras—particularly those installed indoors or in vehicles, which are usually aimed at capturing facial features. The face, though part of the whole body, displays different interactions with global body movements across various action categories and exhibits specific local movements in certain actions. Figure 2 reveals marked differences in facial image sequences and landmark sequences across six action categories related to medical conditions in the NTU RGB+D 120 dataset [2]. Thus, effectively leveraging facial action information can substantially improve action recognition performance under conditions of extensive occlusion.

Human action recognition methods primarily depend on RGB-based [3,4,5,6,7], skeleton-based [8,9,10], RFID-based [11,12,13], or a combination of these approaches [14,15,16]. Recently, 3D CNN-based methods have become increasingly prevalent in RGB-based methods. However, 3D CNNs [17,18] have massive parameters and are vulnerable to failing into local optima during training. To address this issue, some methods [19,20,21] decompose 3D convolutions to reduce the number of parameters. Despite these efforts, these methods use uniform spatial and temporal receptive fields that are inadequate for capturing the nuances of facial movements. For effective facial feature extraction, smaller spatial receptive fields and larger temporal receptive fields are necessary. For the skeleton-based HAR, most methods are based on the whole body skeleton without occlusion. Techniques such as the RA-STAR transformer [22], LART [23], and STGCN [24] attempt to handle occlusion by reconstructing occluded joints. While these methods are robust against partial occlusion, they face challenges with extensive occlusion where reconstructing missing joints becomes impractical. This paper introduces the use of facial landmark sequences for human action recognition under conditions of severe occlusion. Facial keyframes are sparser than full-body keyframes because facial global movements are not consistent across all action categories. The lack of focus on these sparse facial keyframes by existing methods complicates the modeling of global movements in facial landmark sequences.

**Figure 3:**
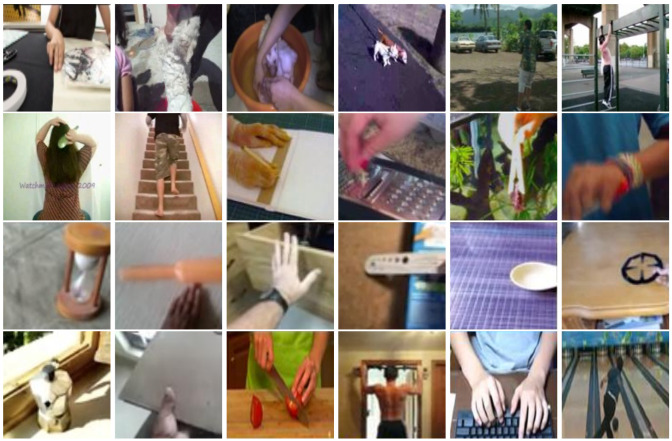
Sample frames of the human action recognition datasets collected from the Internet. The rows show the data from ActionNet, HMDB, Kinetics, something-Something V1, something-Something V2, and UCF.

At present, there are a large number of human action recognition datasets. The UCF101 [25], Kinetics [26], ActionNet [27], HMDB51 [28], Something-something V1, and Something-something V2 [29] were collected from the Internet, as shown in Figure 3. Unfortunately, these datasets are far from the surveillance-like video. The cameras are often too close to the targets, capturing only parts of the targets. Furthermore, most of such actions are strongly related to the background, while the actions in the surveillance-like video are just the reverse. Others, such as ISLD [30], NTU RGB+D [31], and NTU RGB+D 120 [2] datasets, are similar to the surveillance camera setting. These datasets do not contain the occlusions but can be used by adding simulated occlusions [30,32]. In this paper, we consider the surveillance-like environments, such that: (1) The surveillance is frequently installed in the locations where the cameras are easier to capture human faces; (2) The camera viewpoints are far enough to capture the whole scene. Hence, we simulate the intensive occlusion by only using the face data. Some samples of facial image sequences and facial landmark sequences are shown in Figure 2, which are generated from NTU RGB+D and NTU RGB+D 120.

**Figure 4:**
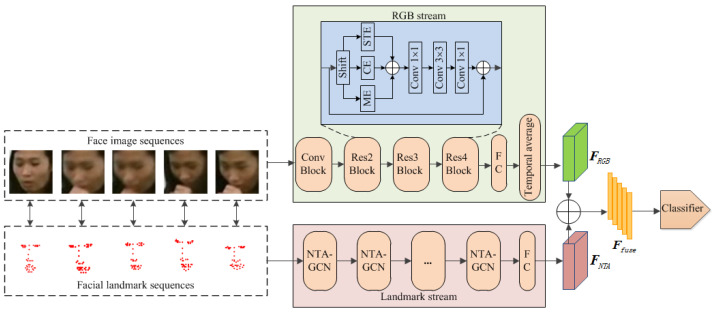
The method of the Face in Action (FIA) method.

Motivated by these observations, we propose Face in Action (FIA), a new two-stream (RGB and landmark) approach for face-based HAR. As shown in Figure 4, the FIA method processes facial image sequences and landmark sequences to classify human actions. Initially, facial images are extracted from videos using bounding box detection, and facial landmark sequences are obtained from the corresponding video coordinates. The RGB stream processes the facial image sequences, while the landmark stream focuses on the landmark sequences. To enhance the modeling of local facial movements, we introduce two key modules: a fine-spatio-multitemporal (FSM) 3D convolution module in the RGB stream and a normalized temporal attention (NTA) module in the NTA-GCN block of the landmark stream. The FSM module employs smaller spatial receptive fields to retain detailed local features and larger temporal receptive fields to capture broader temporal dynamics. The NTA module provides temporal attention, which improves the sensitivity to facial keyframes. Finally, there is a late fusion for the two streams’ outputs.

The following is a summary of the main contributions in this manuscript:

(1) A new approach is presented to human action recognition that excels in scenarios with significant occlusion, utilizing facial action information alone.
(2) A fine-spatio-multitemporal 3D convolution module is developed in the RGB stream. This module employs smaller spatial receptive fields to preserve detailed local facial movements and larger temporal receptive fields to capture extended temporal features.
(3) We introduce a normalized temporal attention module in the landmark stream. This module improves facial keyframe detection through enhanced temporal attention, thereby boosting overall recognition accuracy.

## 2. Related Work

### 2.1. RGB-Based HAR Methods

The performances of RGB-based HAR methods using deep learning have been impressive. Recently, 3D CNN-based methods are frequently employed in RGB-based HAR. For instance, the C3D [33] model utilizes 3D convolutions to directly capture spatio-temporal features. Building on this, more advanced models like R3D [34] and I3D [35] have been developed, leveraging ResNet [36] and GoogleNet [37] architectures, respectively. Despite their effectiveness, 3D CNNs can struggle with local optima due to their high parameter count. To address these challenges, several models have emerged, such as P3D [38], S3D [39], and R2+1D [40]. These approaches replace 3D convolutions with one spatial 2D convolution layer followed by a temporal 1D convolution layer. P3D explores the relationship between spatial and temporal convolutions, while AsyConv [41] refines this by decomposing the spatial convolution into two 1D convolutions. CT-Net [42] further reduces parameter complexity by tensorizing the channel dimension into K sub-dimensions and applying 2D spatial and 1D temporal convolutions within each sub-dimension.Although these methods effectively reduce parameter counts and mitigate local optima issues, they do not necessarily enhance the representational power of 3D convolutions. In contrast, Action-Net [3] employs a different approach by incorporating the action module into ResNet, minimizing reliance on 3D convolutions. Nevertheless, a notable limitation of these approaches is their reliance on fixed-size spatial and temporal receptive fields, which can be inadequate for capturing fine-grained local movements, such as facial expressions.

### 2.2. Skeleton-Based HAR Methods

Currently, the skeleton has garnered widespread attention because skeleton-based methods can ignore the effects of background, illumination, and viewpoint with high-level representations of human body action. Various networks have been used to handle skeleton sequences, such as Recurrent Neural Networks (RNNs), Convolutional Neural Networks (CNNs), and Graph Convolutional Networks (GCNs). GCNs are often used to model the graph-structured data because they have been proven to be effective for processing graph-structured data. GCNs have proven particularly effective for processing graph-structured data. The foundational work by GCN [43] introduced convolution operations designed for graph-structured data, forming the basis of graph convolutional networks. The ST-GCN [44] extended this approach by incorporating temporal dimensions into the graph convolutional method. To delve deeper into the dynamics of human limbs, BPLHM [45] is a graph edge convolutional neural network representing a new edge that integrates its spatial and temporal neighboring edges. The 2s-AGCN [46] proposed the use of varying spatial topologies to distinguish between different action categories. In contrast, SGN [47] utilizes a simpler network architecture to explore joint and temporal semantics explicitly. The CTR-GCN [48] enhances spatial feature extraction by extending dynamic learnable topology to the channel-wise level. Despite these advancements, many of these methods primarily focus on whole-body skeletons and do not address occlusion. Recent approaches have started to tackle occlusion in action recognition. For instance, RA-GCN [32] employs class activation maps to identify key skeleton joints and concentrates on features from joints that are not activated. ActionXPose [30] addresses occlusion by leveraging a pose library, interpolation, and data augmentation techniques. While these methods improve robustness to partial occlusion, they face challenges with intensive occlusion, where recovering missing joints becomes impractical. Additionally, previous GCN-based methods have not placed significant emphasis on temporal attention within skeleton sequence modeling. The facial keyframes are much sparser than whole-body keyframes, so the above methods are harder to model the global movements of facial landmark sequences.

## 3. Method

Our objective is to design a face-based human action recognition method for fully exploiting the facial-action information. As depicted in Figure 4, the FIA—a new two-stream (RGB and landmark) method—is presented. This method processes face image sequences and facial landmark sequences through distinct RGB and landmark streams, respectively. Initially, face image sequences are extracted and cropped from the original videos using a bounding box detector. At the same time, facial landmark sequences are tracked within the same video frames. Consequently, face image sequences focus on capturing detailed local facial movements, while facial landmark sequences provide insights into both global and local facial dynamics. Finally, there is a late fusion for the two streams’ outputs.

**Figure 5:**
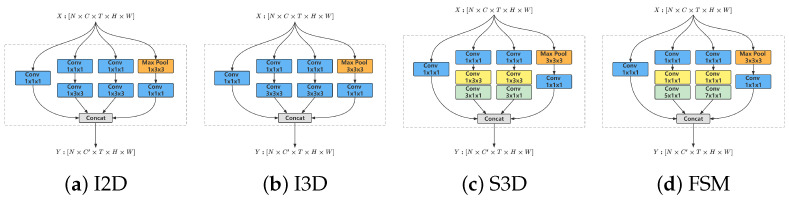
(**a**) 2D Inception module; (**b**) 3D Inception module; (**c**) 3D temporal separable Inception module; (**d**) Fine-spatial-multitemporal 3D convolution module.

**Figure 6:**
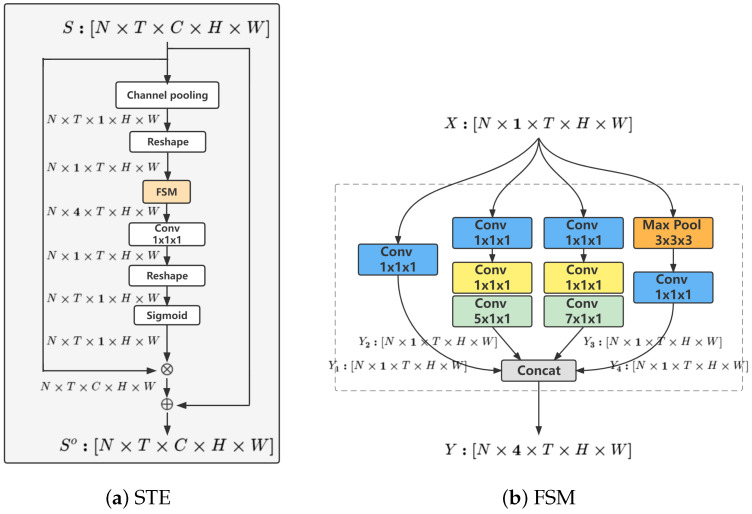
(**a**) The spatio-temporal excitation (STE) of our RGB stream; (**b**) The FSM module.

The FIA’s RGB stream focuses on extracting the facial local movement features from the face image sequences. Traditional 3D convolution modules have limitations in capturing such fine-grained movements. As illustrated in Figure 5, existing 3D convolution models (I2D [37], I3D [35], and S3D [39]) utilize fixed spatial and temporal receptive fields, which are insufficient for modeling detailed facial movements. To address this, we introduce the Fine-Spatio-Multitemporal (FSM) 3D convolution module in the RGB stream. The FSM module is engineered to avoid confusion of local features with surrounding ones and to capture more sparse temporal features. As shown in Figure 4, the RGB stream features a sophisticated 3D convolution network, similar to Action-Net [3] based on ResNet-50 [36]. The spatio-temporal excitation (STE) to which the FSM belongs, channel excitation (CE), movement excitation (ME), and temporal shift operation [49] at the beginning of each residual block. Figure 6 illustrates the spatio-temporal excitation (STE) and the FSM module within it. The implementation details of the FSM are discussed in Section 3.1.

**Figure 7:**
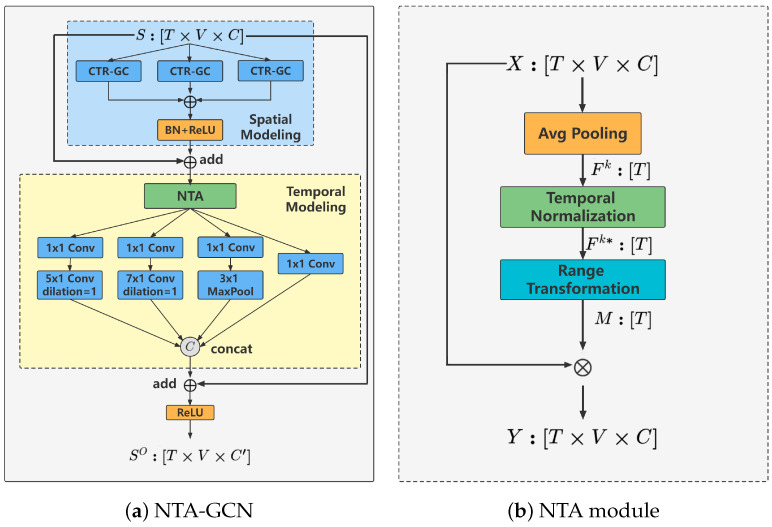
(**a**) The basic block of our landmark stream; (**b**) The NTA module.

The landmark stream of FIA focuses on extracting both global and local movement features from facial landmark sequences. In some action categories, facial keyframes are notably sparser than whole-body keyframes. To effectively capture these sparse facial keyframes, we introduce the Normalized Temporal Attention (NTA) module within the landmark stream. The NTA module produces temporal attention maps that enhance the adaptability and relevance of the facial keyframes. Following this, a powerful GCN is constructed for facial landmark sequences (landmark stream). As illustrated in Figure 7, each basic block of this network, termed NTA-GCN, comprises a spatial modeling module, a temporal modeling module, residual connections [48], and the NTA module. This GCN comprises ten such blocks, followed by global average pooling and a fully connected (FC) classifier for action category prediction, as shown in the landmark stream of Figure 4. Additionally, the temporal dimension is halved by stridden temporal convolution in the $5_{th}$ and $8_{th}$ blocks to optimize computational efficiency and enhance feature extraction. Detailed implementation of the NTA module is discussed in Section 3.2. The specific process is shown in Algorithm 1.
**Algorithm 1** Pseudocode for Human Action Recognition using Facial Cues  1:**Input:** Video sequences with facial images and landmarks  2:**Output:** Action categories  3:Extract facial images and landmarks from the video sequences  4:**for** each frame in the facial image sequence **do**  5:    Apply FSM module to capture local facial movements  6:**end for**  7:**for** each facial landmark sequence **do**  8:    Apply NTA module to detect key facial frames  9:**end for**10:Fuse the outputs from RGB and landmark streams11:Classify the action into one of the predefined categories

### 3.1. Fine-Spatial-Multitemporal 3D Convolution Module (FSM)

The FSM module is designed to efficiently model facial local movements through the use of 3D convolution. It achieves this by reducing the spatial receptive field size to 1×1 and expanding the temporal receptive field to multiple scales. As depicted in Figure 5, the FSM module’s spatial receptive fields are smaller than those in the other three modules, whereas its temporal receptive fields are larger. This design enables the FSM module to effectively capture fine-grained local facial movements and manage large-scale temporal dynamics.

As illustrated in Figure 6b, it has four branches in the FSM. The input $X\in R^{N\times C\times T\times H\times W}$ is fed into the function $f_{i},\left(i=1,\ldots ,4\right)$ to obtain the output $Y_{i}\in R^{N\times \frac{C^{\prime }}{4}\times T\times H\times W}$, where *N* refers to the batch size, *C* and $C^{\prime }$ denote the number of channels, *T* is the number of frames, *H* represents the height, *W* represents the width, and $f_{i}$ denotes the $i_{th}$ branch in the FSM module. Then, $Y_{i}$ are concatenated as total output $Y\in R^{N\times C^{\prime }\times T\times H\times W}$. Meanwhile, it is input to the next layer.

In the FSM module, the 1st and 4th branches employ operations analogous to those in I3D and S3D.

In the 2nd branch of FSM, the X is fed into two 3D convolutional layers $W_{a}$ with kernel size 1×1×1, resulting in

(1) $$\begin{array}{c} X_{a}=W_{a}*X, \end{array}$$

where ∗ denotes the convolution. Then, we model the $X_{a}$, which can be interpreted as

(2) $$\begin{array}{c} Y_{2}=W_{b}*X_{a}, \end{array}$$

where $W_{b}$ is a 5×1×1 3D convolutional layer.

Different from the $2_{nd}$ branch, the $W_{b}$ in the $3_{rd}$ branch is a 7×1×1 3D convolutional layers.

The 1×1 spatial receptive fields in these branches provide precise spatial modeling, ensuring that local features are not overwhelmed by adjacent features. Additionally, the larger temporal receptive fields in the FSM module are designed to capture more extensive temporal patterns, thereby enhancing the module’s ability to model complex temporal dynamics.

### 3.2. Normalized Temporal Attention (NTA) Moudule

The NTA module is designed to efficiently produce temporal attention and enhance facial keyframes with a minimal number of learning parameters. It achieves this by generating a temporal attention mask $M\in R^{T}$, which is applied through element-wise multiplication with the input tensor $S\in R^{T\times V\times C}$, where *V* signifies the number of facial landmarks. As depicted in Figure 7b, the NTA module comprises an average pooling, a temporal normalization, a range transformation, and an element-wise multiplication. Average pooling aggregates features along the temporal dimension, temporal normalization compensates for time-based variations, range transformation adjusts the scale of the features, and element-wise multiplication applies the temporal attention mask to the input tensor, thereby highlighting the most relevant facial keyframes.

Avg Pooling. Given an input tensor $X\in R^{T\times V\times C}$, we first apply average pooling across the spatial dimensions (*V*) and channels (*C*) to produce a global temporal tensor $F^{k}\in R^{T}$.

Temporal Normalization. The tensor $F^{k}$ is then processed through a temporal normalization layer, which normalizes $F^{k}$ as follows:

(3) $$\begin{array}{c} F^{k*}=\frac{F^{k}-F_{\min}^{k}}{F_{\max}^{k}-F_{\min}^{k}}, \end{array}$$

where $F_{\min}^{k}$ and $F_{\max}^{k}$ represent the minimum and maximum values of $F^{k}$, respectively.

Range Transformation. The normalized feature $F^{k*}\in R^{T}$ ranges from 0 to 1. To address the issue of features being lost when multiplied by zero, we use a range transformation to map $F^{k*}$ to a more suitable range. This transformation is defined as follows:

(4) $$\begin{array}{c} M=\sigma (F^{k*})+\beta , \end{array}$$

where σ is the Sigmoid function and β is a learnable bias.

Element-wise Multiplication. The output of the NTA can be calculated as follows:

(5) $$\begin{array}{ccc} Y & = & X⨂M\\ & = & X⨂(\sigma \left(F^{k*}\right)+\beta )\\ & = & X⨂(\sigma \left(\frac{F^{k}-F_{\min}^{k}}{F_{\max}^{k}-F_{\min}^{k}}\right)+\beta ), \end{array}$$

where ⨂ is the time-wise multiplication. After that, the facial keyframes in features S can be enhanced.

Moreover, Formula (4) can be expressed alternatively as follows:

(6) $$\begin{array}{c} M=\sigma (W^{k}F^{k}+b^{k})+\beta , \end{array}$$

where $W^{k}=\frac{1}{F_{\max}^{k}-F_{\min}^{k}}$ and $b^{k}=-\frac{F_{\min}^{k}}{F_{\max}^{k}-F_{\min}^{k}}$. This alternative formulation allows the NTA module to generate parameters *W* and *b* that are tailored to each sample.

## 4. Experimental Section

### 4.1. Datasets

Two datasets, NTU-FACE and NTU-FACE 120, are generated, which are built upon NTU RGB+D [31] and NTU RGB+D 120 [2]. Moreover, they are designed to replicate a surveillance camera environment and contain both face image sequences and facial landmark sequences. The face image sequences are extracted and cropped from the original videos using Faceboxes [50], with each face image uniformly resized to 64×64 pixels. Facial landmark sequences are detected using PIP-Net [51]. Sequences are excluded if the missing rate exceeds 0.5. The missing rate donates the proportion of frames where faces are not detected.

NTU-FACE: This dataset is a comprehensive resource for face-based human action recognition, comprising 48,063 sequences of face images and facial landmarks. It encompasses 60 action categories, with each action performed by 40 actors and captured from three distinct camera views. There are two benchmarks in the dataset: (1) Cross-subject. Training data comes and testing data comes from two groups, each group containing 20 subjects. (2) Cross-view. Training data comes from camera views 2 and 3, and testing data comes from camera view 1.

NTU-FACE 120: In contrast, NTU-FACE 120 adds 46,489 face image sequences and facial landmark sequences based on NTU-FACE. The number of action categories has doubled. A total of 94552 samples in 120 categories are performed by 106 actors, captured from three different cameras. In addition, this dataset contains 32 setups. There are two benchmarks in the dataset: (1) Cross-subject. Training data comes and testing data comes from two groups, each group containing 53 subjects. (2) Cross-setup. Training data come from samples with even setup IDs, and testing data comes from samples with odd setup IDs.

The representativeness of the NTU-FACE and NTU-FACE 120 datasets in practical applications, particularly in surveillance environments, is a critical aspect of their utility. Designed with a variety of actions performed by diverse actors and captured from multiple viewpoints, these datasets emulate real-world surveillance conditions. The inclusion of actions that range from common daily activities to specific gestures relevant to certain scenarios ensures broad coverage of potential behaviors of interest in surveillance settings. Facial actions, which are the focus of these datasets, are less affected by factors such as clothing changes, background variations, and partial occlusions, which are common challenges in surveillance video analysis. The detailed capture of facial landmarks and the corresponding action categories allow for the detection of micro expressions and movements that could indicate emotional states, health conditions, or potential security concerns. In comparison with other datasets used for human action recognition, such as UCF101 [25] and HMDB51 [28], which often feature actions performed in controlled environments with full-body visibility, the NTU-FACE datasets provide a unique focus on facial cues. This focus is particularly beneficial in scenarios where only the upper body or face is visible to surveillance cameras, such as in crowded public spaces or through security feeds with limited angles.

### 4.2. Implementation Details

We conducted our experiments on a single RTX 3090 GPU, featuring 24 GB of video memory. The CPU was an Intel Xeon E5-2620 v4 @ 2.10 GHz, and the system memory was 64 GB DDR4. We used the PyTorch framework for model construction and training, version 1.7.1. Furthermore, we adopted SGD as the optimizer with momentum 0.9 and weight decay 0.0004 for our FIA model. The training epoch is 100. The learning rate is initially configured as 0.1 and decreased by 10 times at epochs 60, 80, and 90. Our data preprocessing steps included cropping facial regions from raw video frames, resizing images to a uniform size, and normalizing them to match the input format of our model. All images were cropped to 224 × 224 pixels.

In terms of training details, we trained each dataset for 100 epochs with data randomized at the end of each epoch. We employed a cross-entropy loss function and evaluated model performance on a validation set after each epoch. Model performance was assessed using accuracy, and we recorded the best performance at the end of each epoch.

### 4.3. Comparison with the SOAT Methods

The FIA, FIA-RGB, and FIA-landmark with the SOTA RGB-based and skeleton-based HAR methods are compared on the NTU-FACE and NTU-FACE 120 datasets in Table 1 and Table 2, respectively. The FIA-RGB donates the RGB stream in FIA, and the FIA landmark donates the landmark stream in FIA. The face image sequences in NTU-FACE and NTU-FACE 120 are very similar to the image sequences in Something-something V1 and Something-something V2 [29] with a part of the body. Therefore, the RGB stream in FIA compares with S3D [39] and Action-Net [3], both of which are SOTA methods on Something-something V1 and Something-something V2 datasets. The landmark stream in FIA compares with the skeleton-based SOTA methods [47,48] in NTU RGB+D [31] and NTU RGB+D 120 [2]. For these methods [3,39,47,48], we strictly follow the training strategies described in their papers. Notably, the inputs to S3D and Action-Net are replaced with 64×64 facial image sequences, and the inputs to SGN [47] and CTR-GCN [48] are replaced with 2D coordinates of 47 facial keypoints.

On the NTU-FACE and NTU-FACE 120 datasets, our presented method outperforms other methods. On the NTU-FACE, our RGB stream in FIA exceeds current SOTA Action-Net by 1.81% and 3.48% on the two benchmarks, respectively. At the same time, our landmark stream outperforms the current state-of-the-art CTR-GCN by 0.07% and 1.02% on the two benchmarks, respectively. In addition, our FIA model outperforms CTR-GCN and Action-Net by 10.55% and 11.58% on the cross-subject benchmark. On the NTU-FACE 120, our RGB stream exceeds the current SOTA Action-Net by 0.02% and 0.60% on the two benchmarks, respectively. At the same time, our landmark stream outperforms current SOTA CTR-GCN by 0.33% and 1.12% on the two benchmarks, respectively. In addition, the FIA model exceeds CTR-GCN and Action-Net by 5.95% and 3.93% on the cross-subject benchmark and 8.18% and 5.19% on the cross-view benchmark.

As shown in Table 1 and Table 2, the incorporation of the FSM and NTA modules into both 2D and 3D CNN baseline models significantly improves the performance across all evaluation metrics. The FSM module, in particular, shows a significant boost in the F1-score, indicating its effectiveness in capturing nuanced facial movements. The NTA module also contributes to higher Precision and Recall, suggesting its importance in detecting key facial frames.

### 4.4. Ablation Study

The FSM and NTA with their configuration on the cross-view benchmark of the NTU-FACE is analyzed in this subsection. Then, we investigate the effects of FSM and NTA in the two-stream model.

Effectiveness of the FSM module: Firstly, we adopt Action-Net [3] as the baseline, which is based on ResNet [36]. It uses the same spatial and temporal receptive fields. For a fair comparison, we only add the FSM to the STE of Action-Net, as illustrated in Figure 4. The obtained numerical results are listed in Table 3. Each column in Table 3 represents a different aspect of the model’s performance. The ‘Δ Params’ column indicates the change in the number of parameters when specific modules such as FSM or NTA are incorporated into the baseline model. A positive value suggests an increase in the number of parameters, while a negative value indicates a decrease. These values are calculated by subtracting the total number of parameters in the baseline model from that in the model with the added module.

Initially, incorporating the 3D temporal separable Inception module into the baseline yields a 0.20% performance improvement. Subsequently, reducing the spatial receptive field size from 3×3 to 1×1 and expanding the temporal receptive field size from 3 to 5 in the $3_{rd}$ branch of the 3D temporal separable Inception block results in a 2.65% performance gain over the baseline. Expanding the temporal receptive field size of the $3_{rd}$ branch to 7 further improves performance by 2.45%, demonstrating that smaller spatial receptive fields and larger temporal receptive fields are more effective for modeling facial local movements. Additionally, reducing the spatial receptive field size in the $2_{nd}$ branch to 1×1 and the temporal receptive field size in the $3_{rd}$ branch to 5 results in a 2.66% improvement over the baseline. Finally, expanding the spatial receptive field sizes to 5 and 7 in the $2_{nd}$ and $3_{rd}$ branches, respectively, while maintaining 1×1 spatial receptive fields (FSM), results in a 3.48% performance gain over the baseline. The FSM provides outperformance without nearly increasing the number of parameters.

**Figure 8:**
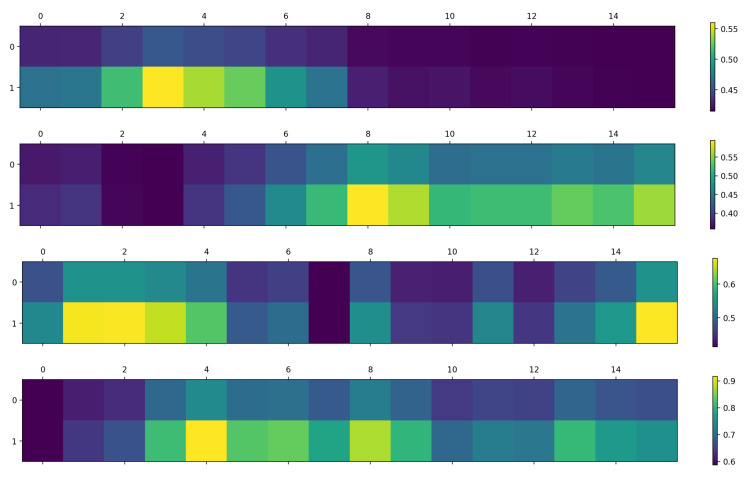
The visualization compares the average features obtained from the temporal attention module using NTA and the without-attended method. The zeroth row shows the average features of sixteen frames without attention, while the first row displays the average features of sixteen frames after applying the NTA module.

Effectiveness of NTA: Table 3 displays the experimental results. Initially, using CTR-GCN [48] as the baseline with a dilation setting of 1, the performance improved by 0.19%. The addition of NTA modules to the basic blocks in the landmark stream resulted in a further performance enhancement of 1.02%. Although the landmark stream increases by only 10 parameters, each basic block within the NTA module adds just one parameter. To more clearly observe the impact of the NTA, we visualize the average features before and after incorporating the NTA module, as illustrated in Figure 8. Figure 8 demonstrates the impact of the Normalized Temporal Attention (NTA) module on the extraction of average features from the facial landmark sequences. The average features are calculated by aggregating the model’s responses to the input data across a sequence of frames, which helps in identifying the model’s focus on specific frames. The zeroth row of the figure shows the average features without the application of the NTA module, indicating a more uniform distribution of attention. When the NTA module is applied, as shown in the first row, there is a noticeable emphasis on certain frames, which are considered key frames for action recognition. This visualization underscores the NTA module’s role in enhancing the model’s ability to detect and focus on these critical frames, thereby improving the overall accuracy of human action recognition. Similar to Table 3, Table 4 illustrates the impact of the NTA module on model performance and parameters. The **Δ Params.** column here also reflects the change in parameters, calculated in the same manner as described above.

Effectiveness of the two modules: For evaluating the contributions of FSM and NTA modules, Table 5 presents a comparison of their individual and combined effects. Baseline models include Action-Net [3] and CTR-GCN [48]. When only the FSM module is added to the RGB stream, it is superior to the baseline by 0.61%. It outperforms the baseline by 0.72% only with the NTA module. When the FSM module and the NTA module work simultaneously, it is superior to the baseline by 0.77%.

Temporal performance evaluation: Table 6 displays the temporal performance results. Our model demonstrated a frame processing time of 35 milliseconds, indicating its capability to handle video streams in real-time. The total inference time for a sequence of 30 frames was measured at 1050 milliseconds, achieving a frame rate of 30 FPS. The latency, which is the time from frame capture to action recognition, was recorded at 50 milliseconds. These results underscore the efficiency of our temporal attention module, highlighting its potential for practical deployment in time-sensitive applications. The selective focus on critical frames reduces computational overhead, enabling rapid decision-making and action recognition.

Performance comparison of fusion strategies. We selected three different fusion strategies for comparison: early fusion, mid-fusion, and late fusion. Each strategy was applied to the same baseline model and tested on the same dataset.The experimental results are shown in Table 7, demonstrating the performance of different fusion strategies in terms of accuracy, processing time, and resource consumption. As shown in Table 7, the late fusion strategy performed best in terms of accuracy, reaching 85.6%, and also showed advantages in processing time and resource consumption. This indicates that the late fusion method can effectively integrate information from different streams while maintaining high processing efficiency and resource utilization.

### 4.5. Comparison with Whole-Body Methods

**Figure 9:**
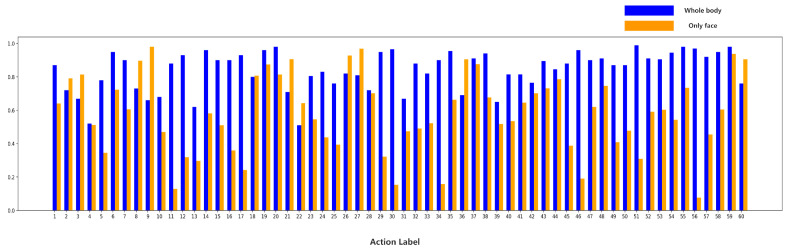
The class accuracy of the whole-body method and our proposed Face in Action (FIA) method on the cross-subject (CS) benchmark of the NTU dataset.

To compare the whole-body method [52] with our Face in Action (FIA) method, we visualize the accuracy for each class on the cross-subject (CS) benchmark of the NTU, as shown in Figure 9. Excitingly, we find that our proposed FIA outperforms the whole-body-based method in 11 out of 60 action categories. Although NTU RGB+D has 60 action categories, there is a low proportion of action categories related to public safety, public health, and state analysis, such as punch/slap, kicking, pushing, wild knife, shoot with gun, sneeze/cough, falling down, headache, chest pain, nausea/vomiting, and yawn, etc. FIA achieves relatively high accuracy on these action categories. In addition, the face occupies a very small spatial area for the whole body. It indicates that it makes sense to HAR by fully exploiting the facial-action information.

Our analysis reveals that the FIA method excels in recognizing categories involving subtle facial expressions and localized movements, such as “cough/sneeze”, “headache”, and “nausea/vomiting”. In these categories, facial information is key, as action details are often concentrated on the face with less distinct or irrelevant movements in other body parts. Moreover, in some action categories like “yawn”, facial movements are the main indicators of the action, while body movements may be subtle or occluded in video footage. The FIA method’s ability to leverage facial information for more robust action recognition largely explains its superior performance in these 11 action categories over whole-body methods.

## 5. Conclusions

A novel two-stream network (FIA) is introduced in this paper for face-based HAR. The fine-spatio-multitemporal (FSM) 3D convolution module and the normalized temporal attention (NTA) module are the key components of the FIA. The FSM in the RGB stream focuses on the modeling of facial local movements. The NTA in the landmark stream pays attention to capturing the sparse facial keyframes. Both mathematical analysis and numerical results on the NTU-FACE and NTU-FACE 120 datasets verify that FIA outperforms other SOTA HAR methods for face-based human action recognition. In addition, FIA outperforms the method with whole-body inputs in 11 out of 60 action categories. Notably, our method is the first to recognize human action by fully exploiting the facial-action information. We believe that our work will influence the development of human action recognition in the future, making it easier to recognize human action in surveillance-like environments. However, our method can be further improved. Our method adopts the late fusion for the two modalities, which does not make use of the complementarity between face image sequences and facial landmark sequences. In the future, we will explore deep fusion for making full use of the complementarity between the two modalities.

## Figures and Tables

**Table 1:** Classification accuracy and parameters comparison against SOTA HAR methods on the NTU-FACE dataset.

Method	RGB	Landmark	NTU-FACE			
			CS (%)	Cross-View (CV) (%)	Parms.	F1-Score
S3D	✓	✗	45.91	46.40	7.96 M	0.77
Action-Net	✓	✗	46.77	42.67	27.85 M	0.78
I3D	✓	✗	47.50	**46.20**	26.9 M	0.80
+FSM	✓	✗	**48.58**	46.15	27.85 M	**0.81**
SGN	✗	✓	45.54	49.07	0.69 M	0.76
CTR-GCN	✗	✓	47.80	56.68	1.49 M	0.75
ResNet-50	✓	✗	47.85	55.56	1.35 M	0.81
+NTA	✗	✓	**47.87**	**57.70**	1.53 M	**0.83**
FIA	✓	✓	**58.35**	**55.69**	29.38 M	**0.84**

**Table 2:** Classification accuracy and parameters comparison against SOTA HAR methods on the NTU-FACE 120 dataset.

Method	RGB	Landmark	NTU-FACE 120			
			Cross-Subject (CS) (%)	CSet (%)	Parms.	F1-Score
S3D	✓	✗	34.94	32.18	8.03 M	0.75
Action-Net	✓	✗	37.41	36.89	27.97 M	0.78
I3D	✓	✗	37.40	37.22	25.9 M	0.77
+FSM	✓	✗	**37.43**	**37.49**	27.97 M	**0.80**
SGN	✗	✓	29.28	29.23	0.72 M	0.79
CTR-GCN	✗	✓	35.49	33.90	1.51 M	0.78
ResNet-50	✓	✗	35.68	34.98	1.53 M	0.81
+NTA	✗	✓	**35.82**	**35.02**	1.54 M	**0.82**
FIA	✓	✓	**41.34**	**42.08**	29.51 M	**0.83**

**Table 3:** Comparisons of FSM validation accuracy and parameters under various settings.

Methods	${branch}_{2}$	${branch}_{3}$	Params.	Acc (%)
Baseline-RGB	-	-	27.85 M	42.67
+S3D	t = 3, s = 3	t = 3, s = 3	27.85 M	42.87
+A	t = 3, s = 3	t = 5, s = 1	27.85 M	45.32
+B	t = 3, s = 3	t = 7, s = 1	27.85 M	45.12
+C	t = 3, s = 1	t = 5, s = 1	27.85 M	45.33
+FSM	t = 5, s = 1	t = 7, s = 1	27.85 M	**46.15**

**Table 4:** Comparison of validation accuracy and parameters for the normalized temporal attention module under various settings.

Methods	Params.	Acc (%)	Δ Params.
Baseline-landmark	1.49 M	56.68	-
dilation = 1	1.53 M	56.87	-
dilation = 1 + NTA	1.53 M	**57.70**	**0.010 K**

**Table 5:** Comparison of validation accuracies and parameters between fine-spatial-multitemporal (FSM) 3D convolution and normalized temporal attention (NTA) modules with the baseline.

Methods	Params.	Acc (%)
Baseline	29.34 M	54.92
Baseline + FSM	29.34 M	55.53
Baseline + NTA	29.38 M	55.64
Baseline + FSM + NTA	29.38 M	55.69

**Table 6:** Temporal performance metrics of the proposed model.

Metric	Description	Value	Unit
Frame Processing Time	Average time to process a single frame	35	ms
Total Inference Time	Time to process a sequence of 30 frames	1050	ms
Frame Rate	Frames processed per second	30	FPS
Latency	Time from frame capture to action recognition	50	ms

**Table 7:** Performance comparison of fusion strategies.

Fusion Strategy	Accuracy (%)	Processing Time (ms)	Resource Consumption
Early Fusion	52.55	2500	High
Mid Fusion	54.18	2200	Medium
Late Fusion	55.69	2000	Low

## Data Availability

The NTU RGB+D dataset and NTU RGB+D 120 dataset used in this work are available at https://rose1.ntu.edu.sg/dataset/actionRecognition/, accessed on 20 September 2024.
